# Institutionalization of evidence-informed practices in healthcare settings

**DOI:** 10.1186/1748-5908-7-112

**Published:** 2012-11-21

**Authors:** Gabriela Novotná, Maureen Dobbins, Joanna Henderson

**Affiliations:** 1 Faculty of Health Sciences, University of Lethbridge, 4401 University Drive, Lethbridge, AB, T1K 3M4, Canada; 2 School of Nursing, McMaster University, 1280 Main Street West, Hamilton, ON, L8S 4L8, Canada; 3 National Collaborating Centre for Methods and Tools and Health Evidence, McMaster University, 1685 Main Street West, Suite 302, Hamilton, ON, L8S 1G5, Canada; 4 Department of Psychiatry, Child, Youth and Family Program, Centre for Addiction and Mental Health, University of Toronto, 80 Workman Way, Toronto, ON, M6J 1H4, Canada

## Abstract

**Background:**

The effective and timely integration of the best available research evidence into healthcare practice has considerable potential to improve the quality of provided care. Knowledge translation (KT) approaches aim to develop, implement, and evaluate strategies to address the research-practice gap. However, most KT research has been directed toward implementation strategies that apply cognitive, behavioral, and, to a lesser extent, organizational theories. In this paper, we discuss the potential of institutional theory to inform KT-related research.

**Discussion:**

Despite significant research, there is still much to learn about how to achieve KT within healthcare systems and practices. Institutional theory, focusing on the processes by which new ideas and concepts become accepted within their institutional environments, holds promise for advancing KT efforts and research. To propose new directions for future KT research, we present some of the main concepts of institutional theory and discuss their application to KT research by outlining how institutionalization of new practices can lead to their ongoing use in organizations. In addition, we discuss the circumstances under which institutionalized practices dissipate and give way to new insights and ideas that can lead to new, more effective practices.

**Summary:**

KT research informed by institutional theory can provide important insights into how knowledge becomes implemented, routinized, and accepted as institutionalized practices. Future KT research should employ both quantitative and qualitative research designs to examine the specifics of sustainability, institutionalization, and deinstitutionalization of practices to enhance our understanding of these complex constructs.

## Background

Knowledge translation (KT) is a complex, multifaceted, and interactive process that involves a variety of stakeholders at various levels: policy, organizational, healthcare team, and individual [1]. Consequently, KT strategies and interventions with health professionals must differ according to the type of evidence being translated, the intended audience (frontline practitioners, health system managers, policy-makers, the general public), and local contexts [2]. In addition, KT strategies, that we define here as overarching designs that outline carefully planned actions – KT interventions, must take into consideration that healthcare organizations are subjected to multiple demands from funders, clients, and professional bodies regarding the quality of care provided [3].

The complex individual, organizational, and system relations of healthcare organizations may result in significant difficulties in implementing research-informed interventions in real-world settings, despite the apparent ease of implementation in well-controlled research environments [4]. Much of the published KT literature has not addressed these multiple levels of decision-making, but rather has examined the determinants of knowledge use at the individual level [5-7]. Given that changes in clinical practice are not solely dependent on the actions of clinicians, but rather require organizational and systems support, KT strategies and interventions targeting only individual-level factors are limited in their capacity to bring about and sustain significant change [8,9].

Organizational responses to the endorsement of innovations by regulatory bodies, established routines, professional roles, and power structures of health services organizations that might influence the use of available research evidence in decision-making, however, remain largely unexplored in KT research within the healthcare sector [10].

To contribute to such developments in KT research, we introduce the main concepts of institutional theory and discuss how this theory [11-15] enhances our understanding of how to achieve stability and ongoing use of newly adopted practices by their institutionalization. We use the concepts of institutional theory to discuss how organizations engage in different types of behavior when responding to external demands for adopting change, including the pressure to adopt new, research-informed practices. In reviewing both the KT and institutional theory literature, we hypothesize that institutional theory has much to offer the KT field with respect to studying how innovations become institutionalized in healthcare organizations through various activities of organizations, professional groups, governments, and regulatory agencies [16-19].

## Discussion

Organizational practices, including those that are supported by research evidence, have to be deeply entrenched in their respective organizational environments in order to produce substantial changes in the way organizations provide services [15]. Among KT researchers, the sustainability of evidence-informed practices, that refers to their continuous use in organizations after their adoption and implementation has been completed, has been emphasized as an ideal outcome of KT strategies [20]. We, however, argue that although sustainability is related to maintaining new practices in organizations, it does not represent the same level of constancy and pervasiveness as their institutionalization. Even though some organizational practices can be sustained in organizations without being institutionalized, for example practices supported by ‘soft’ (temporary) money provided to organizations or by other forms of temporary supports, it is when new practices become highly institutionalized that their integration into daily activities and routines maintains their impact on organizational functioning without the need for additional external interventions [21]. Thus, institutionalization has been defined as “staying power’ or endurance of change that becomes part of everyday activities or normal practices in an organization’ [[20]:167].

### How can institutional theory enhance our understanding of implementation and sustainability of new practices?

Institutional theory provides a lens for viewing organizations as open systems responsive to wider social and cultural forces in their institutional environments [15]. Institutional environments are comprised of regulatory structures made up of social activities, symbols, and material resources referred to as institutions [15]. Created and endorsed through the actions of government agencies, courts, legislators and professions, after becoming established concepts, institutions attain a high degree of resilience and become self-activating in their nature [15]. The persistence of institutionalized practices is related to their becoming accepted as a natural and ‘obvious’ way of doing things [15,22]. Institutionalization, perceived as making new practices normal/routine part of organizational structures, is driven by two, closely related concepts: ‘self-reinforcing feedback dynamics of heightened legitimacy and enhanced ’taken-for-grantedness” [23]. Legitimacy, understood as an assumption that the actions of an entity are desirable, proper, or appropriate within the norms, values, and beliefs that are part of the external environments of organizations [23], is a critical component of institutionalization. Adopting organizational practices that are considered valid and appropriate in a given organizational field makes healthcare organizations appear as competent service providers worth to be granted with ongoing supports [13,15,23]. ‘Taken-for-grantedness’ of organizational practices denotes the patterns and shared conventions that define how things should be done. Because ‘taken-for-grantedness’ of used concepts and practices is related to the degree of institutionalization, weakly institutionalized practices can be subjected to scrutiny and lower support. Accordingly, when activities and procedures associated with new practices move from being ambiguous, unstructured, and unfamiliar to highly routinized, prescribed, and well-understood, the level of their legitimacy and ‘taken-for-grantedness’ becomes very high [21,23].

Legitimacy and ‘taken-for-grantedness’ are positively connected to maintenance of adopted organizational practices—an appealing goal for KT researchers who want to develop and test KT strategies and interventions that would ensure embeddedness and ongoing use of evidence-informed practice (EIP) in organizations. However, KT researchers need to understand that the permanence and self-actualization of institutionalized practices introduces the elements of rigidity, immutability, and resistance that present barriers when new, more effective practices become available. In addition to the understanding how organizations respond to pressure to institutionalize new practices and make them part of their daily routines, KT researchers need to examine the ways deeply institutionalized practices lose their power and become ‘de-institutionalized’ [24]. Accordingly, the examination of the degree of legitimacy and ‘taken-for-grantedness’ of existing organizational practices that are to be replaced by the new ones should be the first step to be taken to ensure that KT interventions are tailored to the potential resistance to new ideas and practices.

Institutional theory in its evolutionary journey toward explaining the elements of conformity of organizations to their external environments progressed through specific stages and has resulted in different concepts being emphasized during those theoretical developments. In the ‘old’ institutionalism, the influence of coalitions and informal structures and the use of power and authority were emphasized as important for instigating change in organizational behavior [25]. Conversely, the theorists of ‘new’ institutionalism have argued that organizations adopt the same organizational practices due to three isomorphic mechanisms—coercive, normative and mimetic [11].

By means of ‘coercive isomorphism,’ regulations and norms are imposed on organizations by formal pressure of governments and other administrative or funding bodies and shape organizations in similar ways [11,12]. Recent research has suggested however that if governments do not implement effective policy setting, monitoring, and rewarding/sanctioning strategies for controlling their requirements on performance of organizations, the effect of the regulations can be very low [26]. An example of a regulative measure that has led to the implementation and sustainability of suggested practices is the mandate given by the Ontario Ministry of Health and Long-Term Care (MoHLTC)-Addiction Program to all ministry-funded addiction treatment agencies in the province to use Admission and Discharge Criteria and Assessment Tools [ADAT] [27]. The main objective was to establish an initial treatment plan that identifies the most appropriate level and intensity of care for a client entering Ontario’s addictions treatment system. Training of clinicians, funded by the Ministry, was conducted by one of the organizations with the provincial mandate in clinical research, treatment, and training. The adoption and use of ADAT resulted in the development of common protocols in the agencies funded by the Ministry across the province, facilitated smoother move of clients between different levels of services, and helped to eliminate duplication in assessment and treatment planning [27]. Regulatory approaches where the government agency mandates the use of EIP through either financial incentives or the use of regulations and accreditation have however been underdeveloped in the addiction treatment services, and most of the EIP implementation strategies include only education, training, and infrastructure building [28].

‘Normative isomorphism,’ dependent on formal education and production of specialists and on the growth of professional networks, can also make organizations increasingly similar [11,29]. However, if organizations do not provide enough structure and stability for new practices, those innovations might dissipate before they become institutionalized without bringing desired changes [15,21]. An example of a low impact of professionalization on adoption and implementation of EIP is the limited effect educational interventions, such as continuing medical education (CME), have on practice. Despite the strong tradition in maintaining licensure and certifications in regulated healthcare professions, CME does not necessarily promote timely translation of EIP into practice if used alone [30]. Miller *et al.*[31] also found that creation of supportive organizational structures is an important factor for implementation and maintenance of new, evidence informed practices. Addiction counsellors who attended training on motivational interviewing [MI] to gain new knowledge and to master MI-related skills ultimately left their organizations if they, due to insufficient organizational supports, were unable to routinely utilize their skills.

Not all sources of change are related to power of authority or professionalization in organizational fields. An important source of adopting similar practices across organizational fields is modeling other more successful organizations (‘mimetic isomorphism’). The relative omnipresence of some organizational practices does not necessarily indicate their effectiveness, but rather the universality of imitating processes [11]. For example, addiction treatment programs affected by mutual-help initiatives in their communities have historically used intensive, abstinence-oriented treatments due to their availability and long tradition in the field [32], even though interventions suggesting more tailored approaches to the severity of alcohol problems, including controlled drinking have been found effective [33]. Those addiction treatment providers had simply emulate other established agencies and continue to use the practices that have been readily available.

All three mechanisms of institutionalization—coercive, normative and mimetic—can proceed across organizational fields and initiate adoption of new practices even if those practices do not make organizations more effective. Hence, practices that are considered legitimate and have become the ‘taken-for-granted’ way of providing services might not necessarily be the most effective practices that are available in organizational fields and vice versa, organizational practices supported by strong research evidence might not be valued as valid treatment options if they do not conform to commonly held values and beliefs of service providers [11].

Little overlap between practices holding a high degree of legitimacy and those with research evidence of treatment efficacy has been observed in the treatment of addictions [31,32]. Garner [34] and Miller *et al.*[35] argue that the addiction treatment sector in the United States (and we suggest in Canada as well) has fallen behind other healthcare sectors in implementing EIP due to its unique historical development. Despite the fact that alcoholism and drug use were framed within a disease model, treatment of addiction has received little support from standard healthcare systems [34]. Instead, services for addiction problems have arisen as treatments guided by personal experiences with addiction and recovery of members of self-help/mutual aid groups and community-led initiatives. Accordingly, those service providers developed a strong preference for particular treatment approaches, despite the lack of research evidence for their efficacy [32]. It has been argued that for some service providers and administrators with lived personal experience of addiction and recovery in Canadian addiction agencies serving women, research evidence supporting efficacy of some treatment approaches does not represent the same level of legitimacy as their positive personal, recovery-related experience with specific treatments [36].

### Organizations engaging in strategic behaviors

‘New’ institutionalism has been very useful in explaining similarity and stability of organizations in a given organizational field [11]. It has, however, failed to attend to the nuances of organizational behaviors, stemming from organizations’ self-interests and active agency [37]. Some organizations attempt to resist external pressure to conform with the requirements of their organizational fields and engage in strategic behaviors that range from complying with the imposed changes to openly resisting them [37]. Those different responses of organizations to external pressures have remained largely unexamined in KT research. In the next section, we discuss how such organizational responses to external conditions impact adoption, implementation, and sustainability of proposed organizational changes.

### Acquiescence

The acquiescence of organizations to their external conditions is related to the degree to which this compliance serves their organizational interests, such as gaining enhanced legitimacy, social supports, and greater stability [37]. The endorsement of new practices by regulations, their inclusion in policy initiatives and strategic plans of federal or provincial authorities, and related financial supports is a strong indication of their socio-political legitimacy [23]. Accordingly, some organizations become consciously compliant with the prerequisites of their funders and administrators and strategically choose to act in accordance with those requirements in anticipation of funding, accreditation, or other forms of support. Regulations that are legally enforced by governments are more effective in making organizations responsive than leaving the adoption and implementation of those practices at the discretion of organizations [37]. Some organizations would implement new practices as a direct response to coercive isomorphism in the form of specific mandates by provincial or federal government agencies [11]. On the basis of those arguments, we suggest the following proposition:

Proposition one: Organizations are more likely to adopt new practices by imitating other organizations or by obeying rules and norms in their environments if newly adopted practices enhance their stature in their organizational field and result in the attainment of additional supports.

This proposition could be tested by using multi-organizations research designs in which interviews and/or surveys with executive directors and managers are conducted to provide information about their motives for compliance with external pressure to adopt new practices.

### Seeking a compromise when responding to external requirements for organizational change

Some organizations might engage in strategic behaviors that represent the necessary compromise between balancing external pressures from the governments and other regulators in their organizational fields and keeping their autonomy. Partial compliance with regulative agencies means that organizations, in order to avoid ‘biting the hand that feeds them,’ will strive to conform to at least minimal standards imposed by their regulators to remain eligible for financial support [37]. Organizations attempting to remain relatively autonomous in their decisions about practices that they want to use become actively engaged in negotiating with governments and funding agencies to reduce the pressure to obey certain policies. In contrast to acquiescence to external regulations, compromising means that organizations comply with external rules to the extent to which they can still remain true to their own interests. Balancing competing requirements of different regulators in the attempts to appease some administrative and funding agencies might be a response of organizations to the multiplicity of external expectations on their functioning. The large number of regulators in a given organizational field with inconsistent requirements on organizational performance negatively affect their compliance with external pressure because it gives the organizations some leeway to maneuver between requirements of those different stakeholders [37]. These developments in institutional theory suggest the following proposition:

Proposition two: Organizations are more likely to make a compromise and adopt new practices if their external environments present consistent requirements on their performance and if those requirements are endorsed by regulations and normative measures.

To find out whether compliance with external pressure is affected by the multiplicity of regulators, KT researchers can conduct environmental scans of organizational fields to develop a list of agencies with regulatory powers in a particular health sector, (*e.g.*, addiction treatment sector). It is important to find out about the mandates, competencies, and measures taken by those regulatory agencies in their effort to endorse the use of EIP. Surveys to examine differences or inconsistencies in the requirements of different regulatory agencies on organizations’ effectiveness, including the use of EIP, could help to test proposition two. Moreover, administrators and managers of healthcare services could be asked to provide information on how the centrality or fragmentation of the organizational fields in which they operate affects their compliance with the requirements of their funders and regulators.

### Avoiding external pressures for organizational change

Oliver [37] suggests that when gains—whether in form of legitimacy or economic efficiency—attainable from new practices are relatively low, organizations avoid adopting such practices. To conceal the non-conformity with external requirements, organizations may engage in ceremonial pretence rather than embrace changes that would lead to better organizational functioning. The dichotomy between appearing as complying with regulations and lack of actual changes in organizational functioning is an important contribution of institutional theory to the understanding of organizational change. Concealment of non-conformity through ‘window dressing’ that involves building organizational structures that would reflect the newly-imposed institutional rules widens a gap between suggested changes and actual work activities [14,37]. In many health sectors, organizations might conceal the avoidance of certain practices by establishing elaborate procedures in the form of committees, task forces, or job descriptions to claim that they responded to external requirements that were endorsed by their regulators. Implementation and the use of new practices, if not closely monitored, might remain low because organizations continue to use treatment approaches that are deeply embedded (institutionalized) in their organizational structures. Periodic occurrence and use of the ‘right’ terminology in many health sectors suggests that organizations are aware of the existence of the innovations; however, the mere acknowledgment of the existence of such practices does not necessarily lead to their implementation and sustainability [11].

Other tactics that are used by organizations to avoid full compliance with external pressure include reducing the extent to which their actions are subjected to evaluation and inspection of their regulators by slightly modifying their organizational goals and activities. Based on these developments in institutional theory, we suggest the following proposition:

Proposition three: Organizations will attempt to disguise their nonconformity with external environments and circumvent the conditions under which they operate to avoid external pressure to adopt new organizational practices.

To test this proposition, we suggest qualitative, exploratory research designs to enhance understanding of the tactics used by organizations to conceal their avoidance of implementation of certain practices. Self-report of employees working in organizations that undergo changes in their practices might not be a sufficient research method to capture whether real change occurred and whether organizations strategically avoid their compliance. Organizational ethnographies and qualitative case studies might effectively capture the processes inside the organizations that allow for concealing the non-compliance.

### Actively opposing changes and/or changing external environments

Two remaining strategic responses—defiance and manipulation—take the form of active resistance by organizations to their external environments [37]. When their internal goals and structures dramatically differ from proposed practices, organizations become defiant by attempting to dismiss the influence of external pressure. For example, some service providers working in addiction agencies serving women with substance use issues across Canada openly dispute practices considered evidence-informed if those practices do not correspond with their own personal beliefs of what constitutes effective recovery [36]. Questioning practices that are not compellingly presented or justified by government agencies or other proponents of change has been frequently observed in organizational research [38]. Institutional theorists assert that organizations will openly dismiss suggested changes if they are confident they will not lose external supports or their legitimacy and that they can ‘survive’ such organizational behavior [11]. Strong commitment of organizations to the practices that they deem rational and easily defendable to regulators can result in challenging imposed changes. Moreover, when organizations’ internal interests substantially depart from values and norms imposed by the new practices, more intense and straightforward rejection of those externally enforced practices are likely to occur through denouncing or belittling both the proposed practices and their proponents [37]. Some addiction service providers who themselves have been in recovery openly question the ability of researchers who do not have any personal experience with addiction or recovery to identify and test effective treatments for individuals with substance use issues [38]. Reflecting on all of these scenarios in organizational strategic behavior, we suggest the following propositions:

Proposition four: Organizations might employ defiant strategies to resist implementation and use of new practices when they believe they have little to lose by diverting from those prescribed practices.

Proposition five: Organizations challenge or attack proposed organizational practices when their internal goals and values substantially differ from those that are endorsed by their environments.

Testing the above propositions will require KT researchers to engage in an open dialogue with organizations that have a strong commitment to certain types of evidence. Using methods in which administrators, managers, and service providers can be engaged in more reflective discussions about the types of evidence they use and the decisions they make in their job positions can bring important insights for KT researchers about the sources of resistance to change. Collaborative or participatory research designs could enhance understanding of the spectrum of beliefs and values of professionals in organizational fields that encompass practices driven by different ideologies and values systems.

Active change of the power relations in organizational fields, referred to as manipulation, is the most active response of organizations to the institutional pressures. Reducing the sources of pressure by inviting influential stakeholders to become involved in organizational structures [37] along with creating voluntary coalitions of organizations driven by mutual benefits are the common tactics used by organizations to avoid change [11]. Coalition-building aims at modifying the external environments in which organizations exist in order to gain approval for the directions that are more in line with their own interests [37]. This type of organizational behavior is most effective in changing the landscape of whole organizational fields as organizations increasingly become in control of new norms and practices and actively redefine what should be considered legitimate choices in treatment. On the basis of these concepts in institutional theory, we suggest the following proposition:

Proposition six: To avoid external pressure to adopt organizational practices that are not congruent with their interests, organizations will attempt to reshape their institutional environments by making coalitions with important stakeholders, influencing beliefs and values in their respective fields, as well as gaining control over the criteria of evaluation of their services.

Conceptualization and testing of this proposition could involve analysis of organizational fields. Using stakeholder influence mapping [39] that focuses on identification of the relationships and influences of key organizations, KT researchers can identify organizations that need to be involved in KT strategies because their ‘buy-in’ is a necessary prerequisite of effective knowledge translation.

Even though the suggested propositions regarding the strategic behaviors of organizations provide insights into institutionalization processes, challenging and deinstitutionalizing the existing practices might be necessary to successfully institutionalize the new ones [24].

### Deinstitutionalization

Practices that are connected with an organization’s core values and have a high level of legitimacy and durability can present a difficult challenge to be overcome by innovators who want to replace them with new insights, ideas, and approaches [21,23]. The conditions under which the established practices can dissipate or erode over time can be explained by deinstitutionalization, a concept that refers to ‘the delegitimization of an established practice or procedure as a result of organizational challenges or the failure of the organization to reproduce previously legitimated or taken-for-granted organizational actions’ [24].

Deinstitutionalization of organizational practices can be preceded by political, functional, and social pressures within and beyond organizations [24]. Increased workforce diversity tends to weaken organizational culture and interrupts the continuation of established organizational activities. Internal conflicts that are accompanied by changes in power relations among different professional groups add to the fragmentation of organizational consensus about what constitutes legitimate organizational behavior [24,40]. Moreover, mounting performance crises contribute to the delegitimization of established organizational practices that no longer represent appropriate and desired actions or products [24]. Those challenges might be heightened by a need to increase technical and functional utility of organizational practices, particularly in the fields in which criteria for efficiency and effectiveness of provided health services have changed substantially. These arguments are the basis for our next proposition:

Proposition seven: Institutionalized status of organizational practices can be significantly weakened by workforce diversity, reallocation of power within organizations, internal conflicts and the declining performance of organizations and related crises.

Examination of some of the variables put forward in this proposition can have substantial impact on implementation and testing of KT strategies and interventions. We argue that KT interventions proposing new practices need to be tailored to the degree of gradual ‘erosion’ of established practices [24]. KT researchers need to examine whether deinstitutionalization of established practices can be affected by strong organizational inertia that might moderate the pace of replacing the ‘old’ practices with new ones. Interviews with administrators and managers who might not want to relinquish the good old practices because they are afraid of the new ones being too disruptive or costly for an organization can help KT researchers understand such inertial tendencies of organizations.

## Summary

Organizations often act strategically when they are expected to adopt new practices and embrace change toward the use of EIP. The purpose of this article was to present propositions grounded in institutional theory that could suggest the possible avenues of future KT research to increase our understanding of such organizational behavior. One of the main arguments we presented is that for new ideas and knowledge to become transformed into routinely used organizational practices, their institutionalization should be an ultimate goal of KT strategies as it represents their incorporation into organizational structures and integration into the regulative, professional, and cultural-cognitive functions of organizations [21]. At the same time, KT researchers need to understand and examine the principles of deinstitutionalization of existing practices to be able to develop and test effective KT strategies that would introduce the new concepts and approaches when they become available. While most of our examples of organizations behaving strategically come from addiction treatment sectors in the United States and in Canada, we believe that concepts of institutional theory reflected in our propositions can be applied across healthcare services. We suggest that future KT research can draw on the presented propositions and examine them empirically in order to predict the conformity or resistance of organizations to KT strategies or interventions to adopt and institutionalize new practices.

Because the concepts of institutional theory that we presented apply to the organizational level of analysis, testing of some of the propositions would require multi-organizational research designs to depict organizational changes toward adopting new practices across a particular organizational field. Quantitative research designs could be used to examine the association between the changes in external environments (government measures, organizational mandates, monitoring strategies or availability of resources/incentives to endorse change) and the actual changes across organizational fields measured at organizational levels.

Given the nature of the data needed for increased understanding of the role of individual actors—either individuals or organizations—in institutionalization processes, KT research would likely benefit from increased use of qualitative approaches [41,42]. Strategic behavior of organizations and the multiple interactions between organizations and their contexts might be more effectively captured in organizational ethnographies and observational, prospective case studies that examine the process-oriented data and account for holistic aspects of the cases [43,44]. Self-report and mono-method research often threatens the validity of research conducted in organizational environment. Accordingly, organizational research conducted as case studies spanning several levels of analysis while relying on real-time data, collected from multiple sources [45] can help identify the mechanisms of institutionalization. Examining how ideas become materialized and embedded in organizations could uncover environmental conditions that are in KT research often acknowledged conceptually but have rarely been examined empirically.

## Competing interests

The authors declare that they have no competing interests.

## Authors' contributions

GN conducted the review of literature on institutional theory and drafted the manuscript. MD and JH reviewed and suggested changes to the manuscript. All authors read and approved the final manuscript.
